# New Advances in Short Peptides: Looking Forward

**DOI:** 10.3390/molecules27113635

**Published:** 2022-06-06

**Authors:** Vasso Apostolopoulos, Joanna Bojarska, Tsun-Thai Chai, Jack Feehan, Krzysztof Kaczmarek, John M. Matsoukas, Octavio Paredes Lopez, Michele Saviano, Mariusz Skwarczynski, Jillian Smith-Carpenter, Mariano Venanzi, Wojciech M. Wolf, Piotr Zielenkiewicz, Zyta M. Ziora

**Affiliations:** 1 Institute for Health and Sport, Victoria University, Melbourne, VIC 3030, Australia; 2 Australian Institute for Musculoskeletal Science (AIMSS), Immunology Program, Melbourne, VIC 3030, Australia; 3 Institute of General and Ecological Chemistry, Faculty of Chemistry, Lodz University of Technology, Zeromskiego 116, 90-924 Lodz, Poland; 4 Department of Chemical Science, Faculty of Science, Universiti Tunku Abdul Rahman, Kampar 31900, Malaysia; 5 Institute of Organic Chemistry, Faculty of Chemistry, Lodz University of Technology, Zeromskiego 116, 90-924 Lodz, Poland; 6 NewDrug PC, Patras Science Park, Platani, 265 04 Patras, Greece; 7 Department of Physiology and Pharmacology, Cumming School of Medicine, University of Calgary, Calgary, AB T2N 4N1, Canada; 8 Center for Research and Advanced Studies of the National Polytechnic Institute, Mexico City 07360, Mexico; 9 Institute of Crystallography (CNR), URT Caserta, Viale A Lincoln 5, 81100 Caserta, Italy; 10 School of Chemistry & Molecular Biosciences, The University of Queensland, St. Lucia, QLD 4072, Australia; 11 Department of Chemistry and Biochemistry, Fairfield University, 1073 N. Benson Rd, Fairfield, CT 06824, USA; 12 PEPSA-LAB, Department of Chemical Science and Technologies, University of Rome, Tor Vergata, 00133 Rome, Italy; 13 Institute of Biochemistry and Biophysics, Polish Academy of Sciences, Pawinskiego 5a, 02-106 Warsaw, Poland; 14 Department of Systems Biology, Institute of Experimental Plant Biology and Biotechnology, University of Warsaw, Miecznikowa 1, 02-096 Warsaw, Poland; 15 Institute for Molecular Bioscience (IMB), The University of Queensland, Saint Lucia, QLD 4072, Australia

It is beyond doubt that short peptides hold significant promise in bio-medicine, as the most versatile molecules, both structurally and functionally. Short peptides do not induce undesired autoimmune responses, have better tumour penetration capacities, as well as the ability to cross cell membranes and the blood–brain barrier. Thus, they have crucial relevance in the treatment of both cancers and neurological disorders. In addition, peptides control cellular functions as versatile bio-messengers, and their influence on rejuvenation of the human body, cell regeneration, and longevity cannot be overlooked. Short peptides are also naturally suited to effectively combat a diverse range of pandemic-prone pathogens, of any origin, regardless of mutation. Cyclopeptides have the internal ability to self-assemble, suitable for the development of complex, tailored drug delivery systems. Peptide nucleic acids are also ideal antisense antibiotics and gene silencers, while peptide aptamers are an appealing alternative to monoclonal antibodies. Novel nano-peptide-based technologies are at the heart of biomedical innovation [1,2,3,4,5,6]. Not surprisingly, there is increasing attention toward studies on these key elements of “life”.

The COVID-19 pandemic has pushed numerous (pre)clinical studies, including bio-evaluation of short peptides, and diverse fields of science and biotechnology have been reshaped, leading to unlimited possibilities and applications of short peptides, for prevention, protection, diagnosis, and therapy, in the future. Indeed, great research efforts are being invested into overcoming the drawbacks of peptides, but further advanced studies are required.

In the face of the dynamic development of studies on short peptides, rapid, open-access publishing of scientific surveys, innovations, new insights, and ideas in one place is required to guarantee transparent availability to a wide readership. This was reflected in the first edition of the Special Issue *Advances in Research of Short Peptides*. The issue received a large number of truly impressive and gratifying contributions, each of which reveals still open problems or challenges [1,7,8,9,10,11,12,13,14,15,16,17,18,19,20,21,22,23,24,25,26,27,28,29,30,31]; therefore, it seems advisable to the Editors to extend a follow-up collection of articles providing the newest results of research on short peptides with a more flexible approach worth being further developed toward applications in bio-medicine. As we move forward to new advances and look back on the studies on short peptides, the aim remains the same—reaching their full potential.

In view of the above, we are pleased to introduce the second edition of the Special Issue *Advances in Research of Short Peptides II*. On behalf of the Editorial Board of the journal *Molecules*, we very much look forward to interacting with potential authors to disseminate their cutting-edge findings through this refreshed academic platform, supported by respected world leaders in science to increase its global profile and recognition. This advanced multidisciplinary forum, for studies on a variety of themes related to short peptides, devoted to science and technology, provides the possibility to promote interaction among academics, researchers, and industry across the globe and exchange research findings.

Some of the most active areas of interest include, but are not limited to, the following:

- Synthesis of modified amino acids, short peptides, and their mimetics;
- Supramolecular aspects of short peptide-derived structures;
- Short peptide engineering and peptide-based nanotechnology;
- Peptide conjugations and modifications, including peptide nucleic acids (PNAs), nucleopeptides, peptide interferons, peptide aptamers, macrocycles, glycopeptides, etc.;
- Bioactive short peptides (nature-/food-derived) and antimicrobial peptides;
- New natural and artificial sources of short peptides (e.g., integrated venomics, peptide-display libraries);
- New computational tools, methods, approaches, databases, as well as technologies for studies on short peptides;
- Peptide–protein interactions;
- Imaging agents with short peptides;
- Short peptides in stem cell research;
- Short peptides for therapy: vaccinology, diagnosis, and treatment of diverse diseases;
- Short peptides in drug design;
- Short peptides in cosmetology and regenerative medicine.

Finally, the impact of short peptides in treating “untreatable” and rare diseases is of special interest.

Overall, this advance in research perspectives is focused on progress towards safe and effective peptide-based bio-therapeutic approaches. The aim is to stimulate researchers for further development following the presented viewpoint.

We hope that this edition will illustrate state-of-the-art knowledge in peptide science, illuminate emerging trends of a field influencing the world in the future and stimulate further research on short peptides to address unmet clinical needs. The most interesting and highly educational contributions will be named “Editor’s choice”, leading to better visibility on the forum.

In this new edition of the Special Issue, we will publish any type of manuscript of high quality, based on both theoretical approaches and experimental explorations, in as much detail as possible, to make it attractive for prospective authors, both experts and young researchers.

Together, we will develop this Special Issue into a respected venue for collecting new advances on short peptides.

Thus, we encourage everyone to disseminate your valuable findings on this global platform by submitting original research papers, up-to-date reviews, short communications, or brief reports at the forefront of research.
